# Modulation of Cell Identity by Modification of Nuclear Pore Complexes

**DOI:** 10.3389/fgene.2019.01301

**Published:** 2020-01-08

**Authors:** Mercè Gomar-Alba, Manuel Mendoza

**Affiliations:** ^1^ Institut de Génétique et de Biologie Moléculaire et Cellulaire, Illkirch, France; ^2^ Centre National de la Recherche Scientifique, Illkirch, France; ^3^ Institut National de la Santé et de la Recherche Médicale, Illkirch, France; ^4^ Université de Strasbourg, Strasbourg, France

**Keywords:** nuclear pore complex, cell differentiation, deacetylase, budding yeast, Hos3

## Abstract

Nuclear pore complexes (NPCs) are protein assemblies that form channels across the nuclear envelope to mediate communication between the nucleus and the cytoplasm. Additionally, NPCs interact with chromatin and influence the position and expression of multiple genes. Interestingly, the composition of NPCs can vary in different cell-types, tissues, and developmental states. Here, we review recent findings suggesting that modifications of NPC composition, including post-translational modifications, play an instructive role in cell fate establishment. In particular, we focus on the role of cell-specific NPC deacetylation in asymmetrically dividing budding yeast, which modulates transport-dependent and transport-independent NPC functions to determine the time of commitment to a new division cycle in daughter cells. By modulating protein localization and gene expression, NPCs are therefore emerging as central regulators of cell identity.

## Introduction

Complex organisms develop through the generation of cellular diversity from a single undifferentiated cell. How are the main cellular components modulated to produce different types of cells? Understanding the answer to this question is one of the fundamental problems in biology. One way to generate different cell types after division is through the partitioning of regulatory molecules to only one of the progeny cells. In the simplest scenario, the asymmetrically partitioned molecule (or “cell fate determinant”) directs transcription of genes that are important for differentiation of the receiving cell (Li, 2013). Much of the knowledge on this topic has come from the study of simple organisms that exhibit basic forms of cell differentiation. One of the best characterized is the budding yeast *Saccharomyces cerevisiae*. This organism divides asymmetrically, giving rise to mother and daughter cells that differ in their identity and behavior. Indeed, newborn daughter cells have different gene expression patterns than their mothers, which affect cell-type-specific processes such as cell separation, mating-type switching, and cell cycle progression (Colman-Lerner et al., 2001; Di Talia et al., 2009; Haber, 2012).

Recent work from our laboratory revealed that in budding yeast, an enzyme that deacetylates nuclear pore complexes acts as a cell fate determinant in daughter cells (Kumar et al., 2018). Nuclear pore complexes (NPCs) are multi-protein assemblies that forms channels in the nuclear envelope thus connecting the nucleus and cytoplasm. We found that deacetylation of NPCs in daughter cells modulates their gene expression program by multiple mechanisms. These findings established that NPCs are biochemically and functionally different in budding yeast mother and daughter cells. Because NPCs are major regulators of nuclear composition and gene expression in eukaryotes, the discovery of cell-type-specific acetylation of NPCs in yeast opens the possibility that similar mechanisms may regulate cell differentiation in multicellular organisms. Here we will briefly describe the structure and function of NPCs, summarize the main mechanisms by which they regulate gene expression and differentiation in yeast and animal cells, and discuss how modulation of NPC acetylation may shape cell identity in eukaryotes.

## Nuclear Pore Complexes: Roles in Nucleo-Cytoplasmic Transport

Since their initial description as components of the “porous layer” in the nuclear envelope of amphibian oocytes (Callan et al., 1949; Callan and Tomlin, 1950), NPCs were proposed to facilitate the transport of molecules between the nucleus and cytoplasm. Structural studies revealed that NPCs are macromolecular assemblies composed of approximately 30–50 different nucleoporins (Nups) that form a channel across the nuclear envelope (NE). The NPC structure is based on an eightfold radial symmetry and contains specific sub-structures (for recent reviews, see Knockenhauer and Schwartz, 2016; Beck and Hurt, 2017; Lin and Hoelz, 2019). These include the central ring, which lays across the NE; the cytoplasmic and nuclear rings, which are anchored at opposite sides of the central ring; and the cytoplasmic filaments and nuclear basket, associated with the cytoplasmic and nuclear rings, respectively. Although this general structure is highly conserved among eukaryotes, NPCs display significant variability across biological species in terms of size and composition, ranging in size from ~60 MDa in yeast to ~90–120 MDa in humans (Maimon et al., 2012; Lin and Hoelz, 2019). As discussed later, some variability in NPC composition is also present between different cell types in yeast and animal cells.

Functionally, NPCs operate as a selective barrier that allows compartmentalization between nucleus and cytoplasm. Small molecules (below approximately 30 KDa in mass, or 3 nm in diameter) such as ions and metabolites can freely diffuse through the NPC in human cells (Mohr et al., 2009). In contrast, transport of larger molecules including most proteins and RNAs requires assistance of specific transport receptors that translocate their cargo through the NPC channel and deliver it to the other side. Transport of most proteins and some RNA species such as tRNA, rRNA, and micro-RNAs is assisted by proteins of the karyopherin family (reviewed in Köhler and Hurt, 2007). Transport directionality is established by cargo release from karyopherins in either the nuclear or cytoplasmic side of the channel, achieved by the Ran GTPase system (Görlich et al., 2003). In contrast, export of messenger RNA (mRNA) is independent of karyopherins and Ran, and involves a dedicated heterodimeric transport receptor (Nxf1/Nxt1 in mammalian cells, and Mtr2/Mex67 in yeast) (Natalizio and Wente, 2013).

## Role of Nuclear Pore Complexes in Genome Organization and Gene Expression

The function of NPCs in transport is linked to gene expression, since the nuclear concentration of transcriptional regulators and the rate of mRNA export are dependent on nucleo-cytoplasmic transport. In addition, NPCs directly impact gene expression by interacting with chromatin. The nuclear periphery plays a key role in the non-random distribution of chromatin inside the nucleus (Akhtar and Gasser, 2007; Cremer and Cremer, 2010) and is generally considered a repressive environment for transcription in yeast and metazoans (Taddei and Gasser, 2012; Steglich et al., 2013). Early visualization of the nuclear membrane showed heterochromatin preferentially associated with the nuclear periphery, with the exception of areas near nuclear pores (Aaronson and Blobel, 1975). These observations led to the idea that association of active genes with NPCs would facilitate the nuclear export of their transcripts, and conversely, that increased transcription may lead to targeting of active genes to nuclear pores. NPCs would therefore shape chromatin spatial organization and act as platforms to couple transcription and mRNA export—the “gene gating” hypothesis (Blobel, 1985). Supporting this idea, yeast genome-wide studies demonstrate that certain Nups and NPC-associated transport factors (e.g., karyopherins) bind preferentially highly transcribed genes (Casolari et al., 2004) and the existence of a vast number of interactions between gene promoters and components of the nuclear pore basket (Schmid et al., 2006).

In yeast, the best-characterized examples of transcriptionally active genes that associate with NPCs are inducible genes, which are highly expressed under specific environmental conditions. Multiple genes, including *GAL1, HXK1, INO1, HSP104,* and *TSA2* localize in the nuclear interior when repressed, and are recruited to the nuclear pores when induced (Brickner and Walter, 2004; Casolari et al., 2004; Casolari et al., 2005; Dieppois et al., 2006; Taddei et al., 2006; Brickner et al., 2007; Ahmed et al., 2010). Specifically, nuclear pore basket nups, such as Nup2, Nup1, Nup60, or Mlp2 are required for perinuclear localization of the active *GAL1* locus (Brickner et al., 2007; Brickner et al., 2016). Other important factors for the association of *GAL1* to NPCs are components of the Spt-Ada-Gcn5 acetyltransferase (SAGA) complex, and the transcription and mRNA export complex 2 (TREX-2) (Casolari et al., 2004; Cabal et al., 2006; Dieppois et al., 2006; Schmid et al., 2006; Luthra et al., 2007; Dultz et al., 2016). Thus, targeting of active genes to NPCs is promoted by basket nups and mRNA elongation and export factors; interestingly, NPC tethering may be mediated by RNA for some but not all active genes (Casolari et al., 2005; Brickner et al., 2007). Additionally, NPC recruitment of inducible yeast genes relies on specific gene recruitment sequences (GRS) in their promoters, which are necessary and sufficient to drive the gene to the NPCs and for their optimal expression (Ahmed et al., 2010). Strikingly, at least some of these genes remain associated with NPCs for several hours after withdrawal of the stimulus and transcriptional repression. This is linked to their faster reactivation upon a second round of induction—a phenomenon known as “transcriptional memory” that requires Nup100 and the histone variant H2A.Z (Brickner et al., 2007; Light et al., 2010).

In animal cells, NPCs have been shown to modulate both chromatin organization and gene expression. As examples of the role of NPCs in chromatin organization, the nuclear basket protein Tpr is required for the exclusion of perinuclear heterochromatin from NPC-associated areas in HeLa cells infected with poliovirus (Krull et al., 2010), influences HIV integration sites by maintaining an open chromatin architecture near the NPC (Lelek et al., 2015; Wong et al., 2015), and promotes the formation and maintenance of senescence-associated heterochromatin foci in the nuclear interior in Ras-induced senescent cells (Boumendil et al., 2019). NPCs modulate gene expression by associating not only with gene promoters, but also with enhancers and super-enhancers to promote enhancer-promoter interactions through chromatin loops (Ibarra et al., 2016; Pascual-Garcia et al., 2017). In animal cells, Nup98 (homologue of yeast Nup100), Nup93, and Nup153 modulate gene expression through binding to chromatin either at the nucleoplasm or at NPCs (Kalverda et al., 2010; Ibarra et al., 2016; Liu et al., 2017; Pascual-Garcia and Capelson, 2019). Moreover, the role of NPCs in transcriptional memory is also conserved in animal cells. Nup98 mediates enhancer-promoter loop formation to ensure faster and higher expression of hormone inducible genes upon repeated activation in Drosophila (Pascual-Garcia et al., 2017), and promotes transcriptional memory after treatment with interferon gamma in human cells (Light et al., 2013). Thus, the role of Nup98 in transcriptional memory is conserved in yeast, flies, and humans (Tan-Wong et al., 2009; Light and Brickner, 2013; D'Urso and Brickner, 2017).

NPC-dependent mechanisms of gene expression involve their interaction with transcription factors (TFs) and histone-modifying enzymes including acetyl-transferases, deacetylases, and ubiquitin-transferases. For example, in human cells exposed to proliferative signals, MYC is recruited to the nuclear pore basket where it interacts with the nups Tpr and Nup153, promoting the formation of a complex that includes the SAGA acetyltransferase component Gcn5, and regulating the expression of mitogen-stimulated genes (Su et al., 2018). In mouse embryonic stem cells, Nup153 represses developmental genes by recruiting the polycomb-repressive complex 1 (PRC1) subunit RING1B, which catalyzes ubiquitination of histone H2A (Jacinto et al., 2015). Finally, in cardiomyocytes, the histone deacetylase HDAC4 interacts with Nup155 at NPCs, and prevents the association of sarcomeric and calcium signaling genes to the NPCs to negatively regulate their expression (Kehat et al., 2011; D'Angelo, 2018).

## Nuclear Pore Plasticity During Cellular Differentiation

Although the overall structure of NPCs is conserved across species and within cell types, recent evidence indicates that NPCs display cell-type specific variability in their protein composition, which in some cases can affect their gene regulatory functions. Early proteomics studies have revealed that the levels of nups including Nup50, Tpr, Nup214, Nup210, Pom121, and Nup37 showed significant variability across cancer cell lines and human tissues (Guan et al., 2000; Cho et al., 2009; Ori et al., 2013). This opened the possibility that tissue-specific expression levels of certain nups could mediate protein transport and/or gene expression changes during development. This may be the case for murine Nup133, which is predominantly expressed in embryonic progenitors and is required for efficient neural differentiation in ESC and neuronal progenitors (Lupu et al., 2008).

Changes in the levels of specific Nups can affect cellular differentiation by regulating the transcription of developmental genes in specific cell types. A well-characterized example is the transmembrane ring NPC component Nup210. The expression of Nup210 is cell-type specific during mouse organogenesis (Olsson et al., 2004). In an *in vitro* myogenic model, Nup210 levels are low in proliferative myoblasts, but increase during myogenic differentiation (D'Angelo et al., 2012). Interestingly, Nup210 depletion inhibits myotube formation. While absence of Nup210 had no detectable defects in protein import or export, it resulted in downregulation of genes involved in myogenesis and other developmental genes (D'Angelo et al., 2012). Nup210 promotes myoblast differentiation through the recruitment to NPCs of Mef2C, a TF key for the regulation of skeletal and cardiac muscle developmental genes at the nuclear periphery (Raices et al., 2017).

Whereas Nup210 levels increase during myogenic differentiation, the levels of the nuclear basket component Nup153 decrease during neural differentiation (Jacinto et al., 2015; Toda et al., 2017). Thus, Nup153 levels correlate with the degree of cellular plasticity, and evidence suggests that Nup153 promotes the maintenance of an undifferentiated cellular state. In mESCs, Nup153 binds to silenced developmental genes at NPCs and also in the nucleoplasm. Loss of Nup153 causes early neuronal differentiation, probably by deficient recruitment of polycomb repressive complexes to developmental genes (Jacinto et al., 2015). Consistent with a role in repressing differentiation, in rat neural progenitor cells, Nup153 is necessary for maintaining the expression of genes distinctive of the neural progenitor cells specific transcriptional program. Nup153 interacts with the neural progenitor TF Sox2 and together, both factors regulate gene expression through their association with promoters and with 3' gene regions, possibly to mediate transcription and repression, respectively (Toda et al., 2017).

Two important conclusions emerge from these studies. First, Nup levels and thus NPCs composition can vary in the different developmental stages. Second, by modulating gene expression through specific nucleoporins, NPCs can either promote or prevent cell differentiation. This has led to the proposal that specialized NPCs with different characteristics may ultimately lead to cell-specific functions (Raices and D'Angelo, 2012). How differences in NPC composition arise during development in animal cells remains unclear.

## Nuclear Pore Deacetylation Regulates Gene Expression in Budding Yeast Daughter Cells

**Figure 1:**
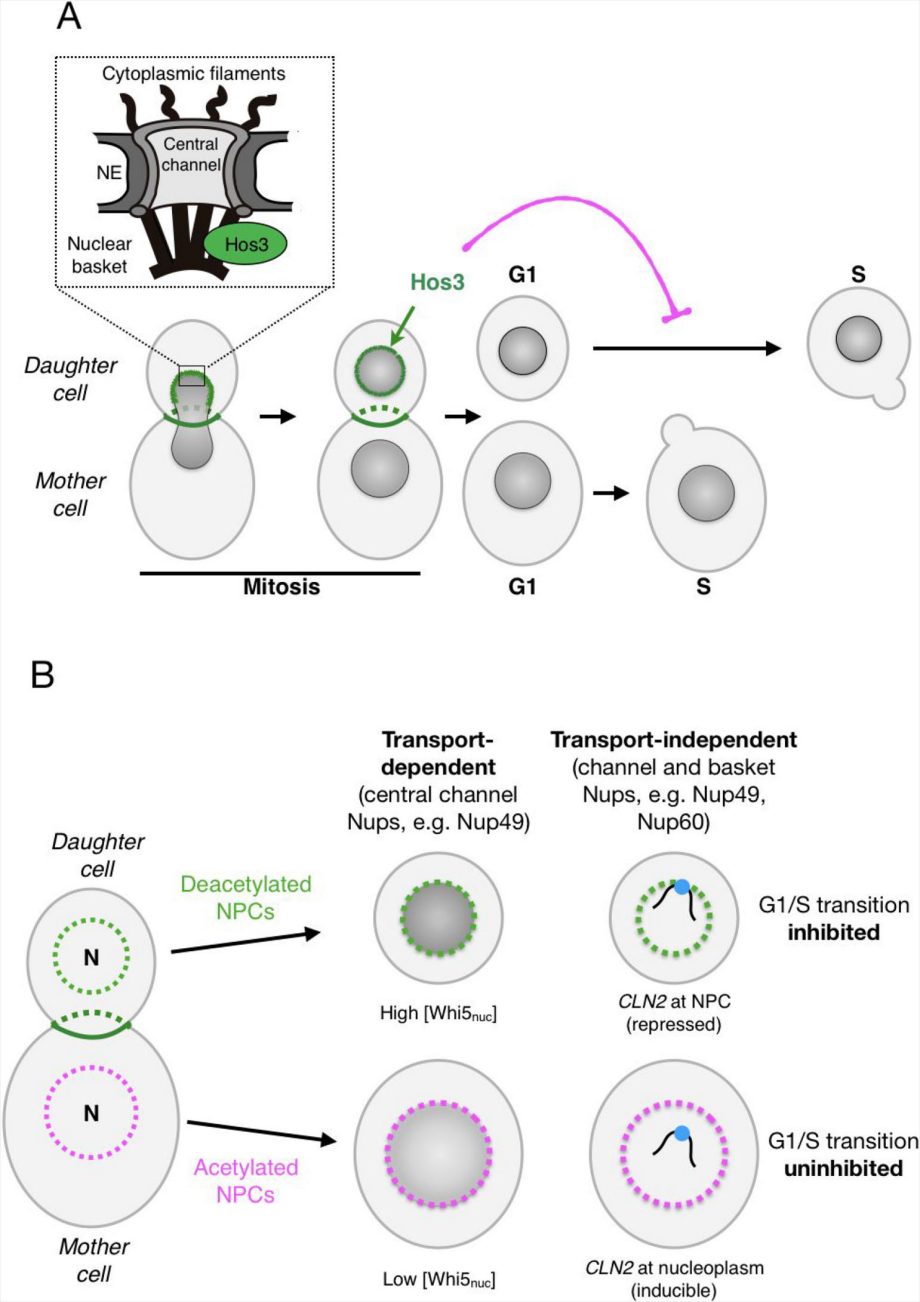
Daughter-cell-specific deacetylation of nuclear pore complexes (NPCs) modulates cell cycle identity in budding yeast. **(A)** During mitotic division, the deacetylase Hos3 (in green) associates with the bud neck and with daughter-cell NPCs during nuclear migration into the bud. Deacetylated NPCs delay the G1/S transition in daughter cells. The inset depicts the main architectural elements of NPCs. **(B)** NPC deacetylation (left) inhibits the G1/S transition in daughter cells through two major mechanisms: nuclear transport of the transcriptional repressor Whi5 (middle) and NPC-mediated repression of the G1/S cyclin gene *CLN2* (right). See text for details.

A new link between changes in NPC composition and cell differentiation was revealed by studies of asymmetric division in budding yeast. Yeast cells proliferate by first growing a bud on the surface of the mother cell; DNA replication is then followed by transport of one of the newly generated nuclei across the mother-bud neck and into the future daughter cell. Cytokinesis produces mother and daughter cells that differ not only in size (at the time of birth, buds are generally smaller than their mothers) but also in their age and transcriptional activity (Li, 2013). This is due to asymmetric partitioning of ageing and cell fate determinants during cell division (Long et al., 1997; Sinclair and Guarente, 1997). The lysine deacetylase Hos3 is a new cell fate determinant that modifies NPCs in newborn daughter cells (**Figure 1A**). Hos3 binds to the daughter-cell side of the septin ring, a cytoskeletal structure that assembles at the bud neck (Wang and Collins, 2014). Hos3 then transiently associates with the periphery of the daughter cell nucleus during anaphase chromosome segregation (Kumar et al., 2018). Since the yeast NE does not disassemble during mitosis, NPCs destined to the bud are in close proximity with the bud neck during anaphase. This suggests that Hos3 transfers from the septin ring to NPCs as they transit through the bud neck. Recruitment of Hos3 to the nuclear periphery is associated with Hos3 association with the nuclear pore basket, and deacetylation of nups at both the central channel (including Nup49, Nup53, and Nup57) and nuclear basket (Nup60). Thus, Hos3 establishes biochemical differences in NPCs between mother and daughter cells: NPCs that are retained in mother cells are acetylated, whereas NPCs transmitted to daughter cells are hypo-acetylated (Kumar et al., 2018). What are the physiological consequences of these differences in NPC acetylation?

Commitment to a new division cycle is regulated asymmetrically in *S. cerevisiae*: daughter cells have a longer G1 phase, and thus start a new cycle later than mother cells. This is due to a cell size-dependent delay that prolongs G1 until daughter cells reach the critical cell size needed to enter in a new round of cell division (Hartwell and Unger, 1977; Turner et al., 2012) and to a size-independent daughter-specific delay of the G1/S transition (Laabs et al., 2003; Di Talia et al., 2009). We found that deacetylation of daughter nucleoporins inhibits cell cycle entry in a manner that is independent of cell size, through regulation of transport-dependent and transport-independent NPC functions in daughter cells (**Figure 1B**). The transport-dependent pathway may act by modulating the nuclear concentration of cell cycle regulators, such as the transcriptional repressor Whi5 (homologue of the retinoblastoma tumor suppressor protein, pRb). The concentration of Whi5 at the start of the cell cycle is highly predictive of G1 duration, and is higher in daughter than in mother cells (Schmoller et al., 2015). We found that higher nuclear concentration of Whi5 in daughter cells requires Hos3-dependent deacetylation of central pore channel nucleoporins (including Nup49) and to a lesser extent, of the basket nup Nup60 in daughter cells (Kumar et al., 2018). How NPC acetylation modulates the nuclear concentration of Whi5 is unclear, but may involve changes in its nuclear transport dynamics. Supporting this possibility, NPC deacetylation reduces the nuclear levels of the karyopherins responsible for Whi5 nuclear import and export (Kap95 and Msn5), raising the possibly that deacetylation of nups in the central channel inhibits their affinity for Whi5 transport receptors. Since Kap95 and Msn5 transport multiple cargoes in addition to Whi5, NPC deacetylation may impact the asymmetric distribution of a plethora of nuclear proteins.

Deacetylation of NPCs also modulates G1 duration independently of nuclear transport. Indeed, deacetylation of the nuclear basket promotes the perinuclear tethering and silencing of at least one key cell cycle control gene, encoding the G1/S cyclin Cln2 (homologue of mammalian Cyclin E). We found that in daughter cells, the *CLN2* locus localizes to the nuclear periphery and associates with the nuclear basket component Nup60 (homologue of mammalian Nup153) during G1, when it is repressed. *CLN2* then moves away from NPCs during S phase, when it is expressed. Artificial targeting of *CLN2* to the nuclear periphery leads to longer G1 phase, suggesting that association with NPCs leads to *CLN2* repression. The daughter-cell-specific recruitment of *CLN2* to NPCs is independent of Whi5 but depends on deacetylation of Nup60 or Nup49 by Hos3 (Kumar et al., 2018). The molecular mechanisms mediating *CLN2* repression at NPCs remain to be elucidated.

**Figure 2:**
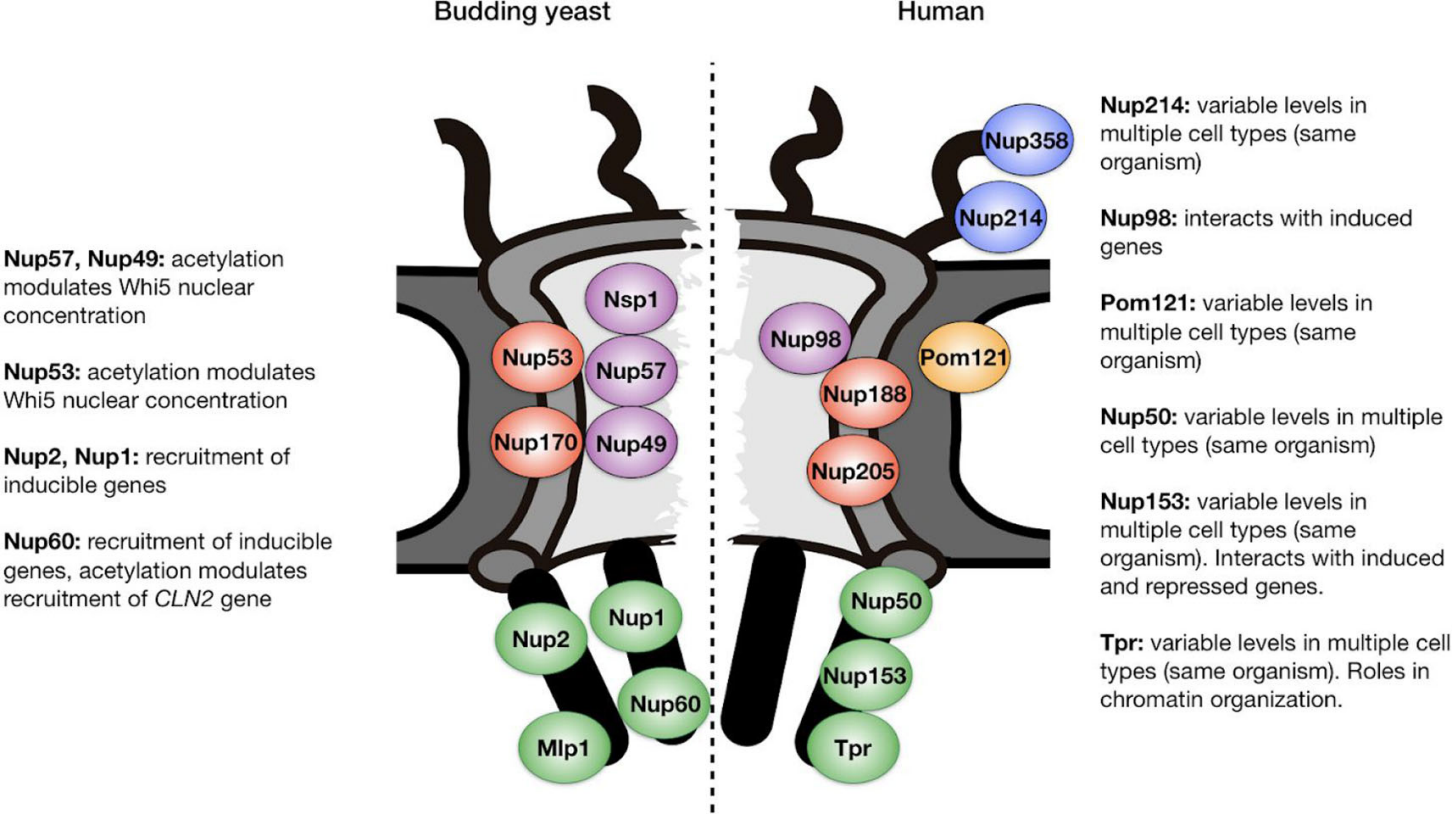
Position within the nuclear pore complex of acetylated nucleoporins in yeast and human cells. Examples of acetylated Nups are based on (Choudhary et al., 2009; Henriksen et al., 2012) and are located in the central channel (purple), inner ring (red), transmembrane ring (orange), cytoplasmic filaments (blue), and nuclear basket (green). Functions relevant to cell identity establishment are annotated. See text for details and references.

In summary, deacetylation of NPCs in daughter cells establishes a key aspect of their identity, inhibiting commitment to a new round of cell division. It is interesting to note that in addition to the putative Hos3 substrates studied so far, additional nups are acetylated in yeast (**Figure 2**); the function of these modifications, and the identity of the responsible acetylases and deacetylases, are not known (Henriksen et al., 2012). Thus, deacetylation of NPCs in yeast daughter cells may have other functions in addition to inhibiting the G1/S transition.

## Future Perspectives

Yeast cells have devised an elaborate mechanism to ensure deacetylation of NPCs in daughter cells and not in mother cells, by coupling inheritance of a Nup deacetylase with passage of the nucleus through the bud neck during mitosis (Kumar et al., 2018). Although this inheritance mechanism may be unique to budding yeast, it remains possible that modulation of NPC acetylation may also impact proliferation and differentiation of animal cells. Although evidence for this is currently lacking, several observations suggest that this possibility warrants investigation. Firstly, as is the case in yeast, human nucleoporins are acetylated, including nups in the central channel (Nup98), nuclear basket (Nup153, Nup50, and Tpr), cytoplasmic filaments (Nup214, Nup358), and inner ring (Nup188, Nup205) (Choudhary et al., 2009; Henriksen et al., 2012) (**Figure 2**). The physiological relevance of these modifications, if any, remains to be explored. Secondly, nucleoporins associate with acetylases and deacetylases both during normal development (Kehat et al., 2011; Su et al., 2018) and in pathological contexts. Indeed, when fused to DNA binding proteins after cancer-induced translocations, nucleoporins such as Nup98 can act as potent transcriptional trans-activators or repressors by recruiting acetylases and deacetylases (Kasper et al., 1999; Bai et al., 2006; Wang et al., 2007).

As we have seen, NPC composition can change during cell differentiation and the mechanisms mediating NPC compositional variability remain unclear (Lupu et al., 2008; D'Angelo et al., 2012; Toda et al., 2017). It is possible that acetylation of specific Nups may affect the ability of NPCs to incorporate additional subunits during development. Nup acetylation may also regulate their ability to interact with gene regulatory factors involved in transcription and/or RNA export. Notably, the ortholog of yeast Nup60 (which is deacetylated to allow for repression of the *CLN2* gene) is Nup153, which as mentioned earlier is essential for repression of developmental genes in rat neural progenitors (Jacinto et al., 2015; Toda et al., 2017). It would be of interest to investigate if Nup153 acetylation or deacetylation is important for its roles in cell fate specification.

Notably, perinuclear deacetylation is important to maintain spatial chromatin organization relative to NPCs, as observed after inhibition of histone deacetylase (HDAC) activity in HeLa cells (Brown et al., 2008). Perinuclear HDACs are thought to regulate gene expression and possibly chromatin-NPC interactions through their well-documented role in deacetylation of histones near promoter regions (Seto and Yoshida, 2014). However, our findings in yeast suggest that nucleoporins may represent a novel category of HDAC substrates with important roles in gene expression and nuclear organization. Interestingly, mammalian deacetylases such as HDAC3 and HDAC4 are enriched in the nuclear periphery and regulate gene expression through regulation of chromatin interactions with the nuclear periphery and/or nuclear pores (Kehat et al., 2011; Poleshko et al., 2017). Identification of the molecular mechanisms by which perinuclear HDACs regulate gene expression, whether by deacetylation of histone or non-histone proteins such as nucleoporins, will be an important future challenge.

## Author Contributions

MG-A and MM wrote the manuscript.

## Funding

This work has benefitted from support provided by the University of Strasbourg Institute for Advanced Study (USIAS) for a Fellowship, within the French national program “Investment for the future” (IdEx-Unistra). Research in our laboratory is supported by grant from “La Ligue Contre le Cancer,” and “Fondation ARC pour la Recherche sur le Cancer” PJA20181208052 to MM, and grant ANR-10-LABX-0030-INRT, which is a French State fund managed by the Agence Nationale de la Recherche under the frame Programme Investissements d'Avenir ANR-10-IDEX-0002-02 to the Institut de Génétique et de Biologie Moléculaire et Cellulaire (IGBMC). MG-A is a recipient of a Postdoctoral Fellowship APOSTD/2017/094 from the Generalitat Valenciana.

## Conflict of Interest

The authors declare that the research was conducted in the absence of any commercial or financial relationships that could be construed as a potential conflict of interest.
